# Supplementary material for: Incorporating human dimensions is associated with better wildlife translocation outcomes
Source: Nat Commun. 2023 Apr 25;14:2119. doi: 10.1038/s41467-023-37534-5 (PMC10130010; doi:10.1038/s41467-023-37534-5)
Supplement: Supplementary file 1 — Supplementary Information [file 41467_2023_37534_MOESM1_ESM.pdf]

Supplementary Information

Table S1. Hypotheses with predictions and references for how the inclusion of human dimensions will vary in translocation efforts.

**Hypothesis**	**Prediction**	**References**
Human dimensions are considered critical for the translocation success of larger and wider ranging taxa	Translocations of mammals and birds will have a higher probability of including human dimension objectives	[1,2]
Human dimensions are thought to be important for the translocation success of species with direct human threats	Translocations of species with threats directly attributed to humans in the IUCN Red List of Threatened Species Database will have a higher probability of including human dimension objectives	[3,4]
The inclusion of human dimensions in wildlife translocations is higher at sties with previous human-wildlife conflict	Translocations in sites where human-wildlife conflict was noted for the species of interest will have a higher probability of including human dimension objectives	[5]
The inclusion of human dimensions for wildlife translocation is higher when local stakeholders are active participants in the project	Translocation case studies that had participation from local stakeholders will have a higher probability of including human dimension objectives	[6,7]

Table S2. Example excerpts from IUCN Re-Introduction Global Perspectives Series case studies that were used to determine the outcome (i.e., survival, reproduction, population increase, high mortality, or extinction) of the project[8–10]. Excerpts that indicated any widespread survival, reproduction, and/or population increase were assigned a positive outcome, and excerpts that indicated either high mortality or extinction were assigned a negative outcome.

**Outcome**	**Examples**	**Binary Outcome**
Widespread Survival	“Approximately 30 of the original founders continue to use the feeders daily, therefore survival has been high with at least 66% of birds accounted for eight years after reintroduction”<br><br>”Founders have survived, are in good condition, and there has been a mean population weight gain, indicating that tuatara can survive and there are sufficient resources at Cape Sanctuary.”	

	”Post release survival has been high (87% for 2012 release and 100% for 2013 release as of August 2014).”	Positive
Reproduction	“Presently, the breeding center has about 50 pudus, and the reintroduction area has ~15 mature individuals that breed every year…”<br><br>“Released agoutis became independent from food supply, experienced high survival rates, and breeding was observed before 12 months after release.”<br><br>“After the third year, stickleback were regularly seen in most of the shallow habitat and found in minnow traps throughout the lake, including various stages of development, with clear signs of high reproductive success.”	
Population Increase	“Increase of rhea population from less than 30 to over 50 in Patagonia National Park.”<br><br>“A self-sustaining population was successfully established; in 2017, five years post-release, 67 individual LAPM (35/ha) were trapped at the release and many (76.1%, n = 51) were in reproductive condition.”<br><br>“Bison have been reintroduced and the population is disease free, genetically diverse, and has grown to 866 individuals representing one of the largest conservation herds in existence.”	
High Mortality	“Of all individuals released in 2007, which had not been subjected to preadaptation period, only about 20% survived the first three months of the critical post-release period, with starvation, predation by puma (Puma concolor) and individuals caught in wire fences being the most frequent causes of death.”<br><br>“Eleven of the 13 translocated lions died after living a mean of 272 ±63 days postrelease, one lost its collar (i.e., it may or may not have died) after just 15 days, and one remains active (released in June 2016).”<br><br>“Poor survival and/or retention of re-introduced aplomados likely due to drought, shrub encroachment, inadequate prey populations, and predators.”	Negative
Extinction	“Mallee emuwren occupancy declined following releases and by July 2019, no birds could be detected at the NCP release sites or surrounding habitat.”	

	"Despite broader monitoring across its former range, including an occupancy study where triplicate surveys of 32 former and reintroduction sites were carried out in November and December 2018, the species has not been detected in the Basin since 2015."<br><br>"By 2010, two years after the restoration effort ended due to the tragic death of the project leader (Kevin Honness), there was scant evidence of swift foxes on BRR and environs."	

Table S3. Pairwise differences, standard error, and *p* values from pairwise comparisons following univariate, two-sided, logistic regression models for taxonomic groups (Amphibian, n = 27; Bird, n = 77; Fish, n = 40; Reptile, n = 40; Mammal, n = 121) with a binary response variable for the inclusion of human dimensions.

	Pairwise Difference	Standard Error	*p* Value
Amphibian - Bird	-1.40	0.59	**0.02**
Amphibian - Fish	-1.02	0.64	0.11
Bird - Fish	0.39	0.41	0.34
Amphibian - Mammal	-1.87	0.57	**< 0.01**
Bird - Mammal	-0.46	0.29	0.12
Fish - Mammal	-0.85	0.38	**0.03**
Amphibian - Reptile	-1.13	0.64	0.08
Bird – Reptile	0.23	0.40	0.49
Fish – Reptile	-0.11	0.47	0.81
Mammal – Reptile	0.73	0.38	0.05

Table S4. Pairwise differences, standard error, and *p* values from pairwise comparisons following univariate, two-sided, logistic regression models for the groups involved in the restoration (Academic, n = 146; Government, n = 228; Local Community, n = 64; Non-profit, n = 158; Private Company, n = 24; Private Landowner, n = 44; Zoo, n = 77) with a binary response variable for the inclusion of human dimensions.

	Pairwise Difference	Standard Error	*p* Value

Academic - Government	-0.12	0.22	0.59
Academic – Local Community	-1.02	0.64	0.11
Academic – Non-profit	-0.29	0.23	0.21
Academic – Private Company	-0.41	0.44	0.35
Academic – Private Landowner	-1.12	0.37	**< 0.01**
Government – Local Community	-0.81	0.29	**< 0.01**
Government – Non-profit	-0.17	0.21	0.40
Government – Private Company	-0.30	0.43	0.49
Government – Private Landowner	-1.06	0.35	**< 0.01**
Government – Zoo	0.32	0.27	0.25
Local Community – Non-profit	0.68	0.21	**0.03**
Local Community – Private Company	0.51	0.48	0.29
Local Community – Private Landowner	-0.25	0.41	0.54
Local Community - Zoo	1.27	0.35	**< 0.01**
Non-profit – Private Company	-0.13	0.44	0.77
Non-profit – Private Landowner	-0.89	0.36	**0.01**
Non-profit – Zoo	0.49	0.29	0.09
Private Company – Private Landowner	-0.76	0.52	0.14
Private Company – Zoo	0.61	0.47	0.19
Private Landowner – Zoo	1.37	0.40	< 0.01

Table S5. Pairwise differences, standard error, and *p* values from pairwise comparisons following univariate, two-sided, logistic regression models for the IUCN threats (Agriculture or Aquaculture, n = 178; Climate Change or Severe Weather, n = 99; Energy Production or Mining, n = 60; Geological Events, n = 2; Human Intrusion and Disturbance, n = 61; Invasives or Disease, n = 143; Natural System Modifications, n = 112; Pollution, n = 83; Residential or Commercial Development, n = 101; Transportation and Service Corridors, n = 78, Biological Resource Use, n = 196) facing each species with a binary response variable for the inclusion of human dimensions.

	Pairwise Difference	Standard Error	*p* Value
Agriculture or Aquaculture – Climate Change or Severe Weather	0.08	0.21	0.60
Agriculture or Aquaculture – Energy Production or Mining	-0.04	0.30	0.82
Agriculture or Aquaculture – Geological Events	0.02	1.42	0.98
Agriculture or Aquaculture – Human Intrusion and Disturbance	0.38	0.30	0.20
Agriculture or Aquaculture – Invasives or Disease	0.61	0.23	**0.01**
Agriculture or Aquaculture – Natural System Modifications	0.57	0.25	**0.02**
Agriculture or Aquaculture – Pollution	0.54	0.27	0.08
Agriculture or Aquaculture – Residential or Commercial Development	0.28	0.25	0.26
Agriculture or Aquaculture – Transportation and Resource Corridors	-0.02	0.27	0.91
Agriculture or Aquaculture – Biological Resource Use	0.08	0.20	0.69
Biological Resource Use – Climate Change or Severe Weather	0.59	0.26	**0.02**
Biological Resource Use – Energy Production or Mining	-0.13	0.30	0.67
Biological Resource Use – Geological Events	-0.06	1.42	0.96
Biological Resource Use – Human Intrusion and Disturbance	0.30	0.29	0.31
Biological Resource Use – Invasives or Disease	0.53	0.23	**0.02**
Biological Resource Use – Natural System Modifications	0.49	0.24	**0.05**
Biological Resource Use – Pollution	0.45	0.27	0.09
Biological Resource Use – Residential and Commercial Development	0.19	0.25	0.42
Biological Resource Use – Transportation and Service Corridors	-0.11	0.27	0.67
Climate Change or Severe Weather – Energy Production or Mining	-0.71	0.33	**0.03**
Climate Change or Severe Weather – Geological Events	-0.65	1.43	0.65
Climate Change or Severe Weather – Human Intrusion and Disturbance	-0.28	0.34	0.40

Climate Change or Severe Weather – Invasives or Disease	-0.06	0.27	0.83
Climate Change or Severe Weather – Natural System Modifications	-0.09	0.28	0.73
Climate Change or Severe Weather – Pollution	-0.13	0.31	0.67
Climate Change or Severe Weather – Residential and Commercial Development	-0.39	0.29	0.18
Climate Change or Severe Weather – Transportation and Service Corridors	-0.69	0.31	**0.02**
Energy Production or Mining – Geological Events	0.06	1.43	0.96
Energy Production or Mining – Invasives or Disease	0.66	0.31	**0.04**
Energy Production or Mining – Natural System Modification	0.62	0.32	0.06
Energy Production or Mining – Pollution	0.58	0.34	0.09
Energy Production or Mining – Residential and Commercial Development	0.33	0.33	0.32
Energy Production or Mining – Transportation and Service Corridors	0.02	0.34	0.96
Geological Events – Human Intrusion and Disturbance	0.37	1.43	0.80
Geological Events – Invasives or Disease	0.59	1.43	0.68
Geological Events – Natural System Modifications	0.55	1.43	0.70
Geological Events – Pollution	0.52	1.43	0.72
Geological Events – Residential and Commercial Development	0.26	1.43	0.86
Geological Events – Transportation and Service Corridors	-0.05	1.43	0.97
Human Intrusion and Disturbance – Invasives or Disease	-11.97	324.7	0.97
Human Intrusion and Disturbance – Natural System Modifications	0.18	0.33	0.57
Human Intrusion and Disturbance – Pollution	0.15	0.35	0.66
Human Intrusion and Disturbance – Residential and Commercial Development	-0.11	0.33	0.75
Human Intrusion and Disturbance – Transportation and Service Corridors	-0.42	0.35	0.23
Invasives or Disease – Natural System Modifications	-0.04	0.26	0.88
Invasives or Disease – Pollution	-0.07	0.29	0.80

Invasives or Disease – Transportation and Service Corridors	-0.64	0.29	**0.03**
Invasives or Disease – Residential and Commercial Development	-0.33	0.26	0.21
Natural System Modifications – Pollution	-0.03	0.30	0.92
Natural System Modifications – Residential and Commercial Development	-0.29	0.28	0.30
Natural System Modifications – Transportation and Service Corridors	-0.60	0.30	**0.05**
Pollution – Residential and Commercial Development	-0.26	0.30	0.40
Pollution – Transportation and Service Corridors	-0.57	0.32	0.07
Residential and Commercial Development - Transportation and Service Corridors	-0.31	0.30	0.30

Table S6. Coefficient estimate, confidence intervals, and *p* values from a two-sided, logistic regression including taxonomic groups, the presence of a local history of conflict with the translocated species (local history of conflict, n = 69), the inclusion of local community groups (local community involved, n = 64), and the presence of direct human threats (presence of a direct human threat, n = 249) with a binary response variable for the inclusion of objectives related to human dimensions.

	Coefficient Estimate	Confidence Interval	*p* Value
Taxonomic Group (Bird)	1.11	-0.03 – 2.47	0.07
Taxonomic Group (Fish)	0.62	-0.66 – 2.06	0.36
Taxonomic Group (Mammal)	1.53	0.43 – 2.86	**0.01**
Taxonomic Group (Reptile)	0.97	-0.29 – 2.41	0.15
Presence of Conflict	1.08	0.47 – 1.71	**<0.01**
Involvement of Local Community Groups	1.49	0.85 – 2.15	**<0.01**
Presence of Direct Human Threats	0.63	-0.08 – 1.38	0.09

Supplementary References

1. Carter, N. H. & Linnell, J. D. C. Co-Adaptation Is Key to Coexisting with Large Carnivores. *Trends Ecol. Evol.* **31**, 575–578 (2016).
2. Dayer, A. A., Barnes, J. C., Dietsch, A. M., Keating, J. M. & Naves, L. C. Advancing scientific knowledge and conservation of birds through inclusion of conservation social sciences in the American Ornithological Society. *Condor* **122**, 1–6 (2020).

3. Manfredo, M. J., Berl, R. E. W., Teel, T. L. & Bruskotter, J. T. Bringing social values to wildlife conservation decisions. *Front. Ecol. Environ.* 1–8 (2021). doi:10.1002/fee.2356
4. Crees, J. J. *et al.* A comparative approach to assess drivers of success in mammalian conservation recovery programs. *Conserv. Biol.* **30**, 694–705 (2016).
5. Madden, F. Creating coexistence between humans and wildlife: Global perspectives on local efforts to address Human–Wildlife conflict. *Hum. Dimens. Wildl.* **9**, 247–257 (2004).
6. Redpath, S. M. *et al.* Don't forget to look down – collaborative approaches to predator conservation. *Biol. Rev.* **92**, 2157–2163 (2017).
7. Doyle-Capitman, C. E., Decker, D. J. & Jacobson, C. A. Toward a model for local stakeholder participation in landscape-level wildlife conservation. *Hum. Dimens. Wildl.* **23**, 375–390 (2018).
8. Soorae, P. S. *Global Re-Introduction Perspectives, 2016: Case-Studies from Around the Globe*. (2016).
9. Soorae, P. S. *Global Re-Introduction Perspectives 2018: Case-Studies from Around the Globe*. (2018).
10. Soorae, P. S. *Global conservation translocation perspectives : 2021*. (2021).
