# Supplementary material for: Incorporating human dimensions is associated with better wildlife translocation outcomes
Source: Nat Commun. 2023 Apr 25;14:2119. doi: 10.1038/s41467-023-37534-5 (PMC10130010; doi:10.1038/s41467-023-37534-5)
Supplement: Supplementary file 3 — Reporting Summary [file 41467_2023_37534_MOESM3_ESM.pdf]

nature portfolio

Corresponding author(s): Mitchell Serota

Last updated by author(s): Feb 20, 2023

Reporting Summary

Nature Portfolio wishes to improve the reproducibility of the work that we publish. This form provides structure for consistency and transparency in reporting. For further information on Nature Portfolio policies, see our Editorial Policies and the Editorial Policy Checklist.

Statistics

For all statistical analyses, confirm that the following items are present in the figure legend, table legend, main text, or Methods section.

n/a	Confirmed	
☐	☒	The exact sample size (*n*) for each experimental group/condition, given as a discrete number and unit of measurement
☒	☐	A statement on whether measurements were taken from distinct samples or whether the same sample was measured repeatedly
☐	☒	The statistical test(s) used AND whether they are one- or two-sided<br>*Only common tests should be described solely by name; describe more complex techniques in the Methods section.*
☐	☒	A description of all covariates tested
☐	☒	A description of any assumptions or corrections, such as tests of normality and adjustment for multiple comparisons
☐	☒	A full description of the statistical parameters including central tendency (e.g. means) or other basic estimates (e.g. regression coefficient) AND variation (e.g. standard deviation) or associated estimates of uncertainty (e.g. confidence intervals)
☐	☒	For null hypothesis testing, the test statistic (e.g. *F*, *t*, *r*) with confidence intervals, effect sizes, degrees of freedom and *P* value noted<br>*Give P values as exact values whenever suitable.*
☒	☐	For Bayesian analysis, information on the choice of priors and Markov chain Monte Carlo settings
☒	☐	For hierarchical and complex designs, identification of the appropriate level for tests and full reporting of outcomes
☒	☐	Estimates of effect sizes (e.g. Cohen's *d*, Pearson's *r*), indicating how they were calculated

Our web collection on statistics for biologists contains articles on many of the points above.

Software and code

Policy information about availability of computer code

Data collection	No code was used for the data collection of this project.
Data analysis	To analyze the data we used R Version 4.0.2. All analyses were preformed by using functions available in base R. Plotting was done using the package ggplot2.

For manuscripts utilizing custom algorithms or software that are central to the research but not yet described in published literature, software must be made available to editors and reviewers. We strongly encourage code deposition in a community repository (e.g. GitHub). See the Nature Portfolio guidelines for submitting code & software for further information.

Data

Policy information about availability of data

All manuscripts must include a data availability statement. This statement should provide the following information, where applicable:

- Accession codes, unique identifiers, or web links for publicly available datasets
- A description of any restrictions on data availability
- For clinical datasets or third party data, please ensure that the statement adheres to our policy

The dataset made from the IUCN Global Re-Introduction Perspective Series has been permanently archived with Zenodo (DOI: 10.5281/zenodo.7086487). The IUCN Red List of Threatened Species and the IUCN Global Re-Introduction Perspective Series can be found at iucnredlist.org and iucn.org. Source data are provided with this paper.

Human research participants

Policy information about studies involving human research participants and Sex and Gender in Research.

Reporting on sex and gender	N/A
Population characteristics	N/A
Recruitment	N/A
Ethics oversight	N/A

Note that full information on the approval of the study protocol must also be provided in the manuscript.

Field-specific reporting

Please select the one below that is the best fit for your research. If you are not sure, read the appropriate sections before making your selection.

☐ Life sciences ☐ Behavioural & social sciences ☒ Ecological, evolutionary & environmental sciences

For a reference copy of the document with all sections, see nature.com/documents/nr-reporting-summary-flat.pdf

Ecological, evolutionary & environmental sciences study design

All studies must disclose on these points even when the disclosure is negative.

Study description	We reviewed 305 case studies of wildlife translocations from the International Union for Conservation of Nature's (IUCN) Global Re-Introduction Perspective Series to analyze how the inclusion of human dimension objectives impacts translocation success. We then identified the primary factors influencing whether translocation efforts incorporated human dimension objectives (e.g. taxa and organization(s) involved). Finally, we tested whether the inclusion of human dimension objectives for translocations has changed over time.
Research sample	We reviewed case studies from the IUCN Global Re-Introduction Perspective Series (2008, 2010, 2011, 2013, 2016, 2018, 2021). For our dataset, we only included case studies of vertebrates (n = 305).
Sampling strategy	Sample size was determined based on data availability. We restricted our case studies to the Global Re-Introduction Perspective Series to maintain a standardization among samples.
Data collection	All authors systematically read through each case study and extracted relevant information for the study. For each case study, we evaluated whether each section in the report (Goals, Success Indicators, Major Difficulties Faced, and Major Lessons Learned) contained information related to the human dimensions of the translocation. We defined the inclusion of human dimension objectives during the planning process as a binary yes/no. We then used our review to identify the key ways in which case studies incorporated human dimension objectives. In addition, we recorded the location, start year of the project, the groups or stakeholders involved in the restoration, the threats to that species, and whether there was a history of conflict between that species and humans in the translocation area. The group(s) or stakeholder(s) involved in the translocation were classified as government, academic, zoo, non-profit, local community, private landowner, and private company. The threats to each species were classified according to the IUCN Red List of Threatened Species Database and included residential & commercial development, agriculture & aquaculture, energy production & mining, transportation & service corridors, biological resource use, human intrusion and disturbance, natural systems modification, invasive & other problematic species, genes, & diseases, pollution, geological events, and climate change & severe weather. Finally, using the Project Summary and Reason(s) for Success sections of the reports, we classified the outcome of the project as positive or negative based on the outcome to the translocated wildlife population. Case studies with a positive outcome reported widespread survival, reproduction, or population growth, whereas case studies with a negative outcome reported either widespread mortality or population extinction.
Timing and spatial scale	We reviewed case studies from May 2021 to October 2021. Case studies from the Global Re-Introduction Perspective Series are from around the world - North America (n = 69) and Asia (n = 67), Oceania (n =56), Europe (n = 53), Africa (n = 35), and South America (n = 25). In addition, case studies range from a start date of 1922 to 2018.
Data exclusions	Our analysis only looked at the translocations of mammals, birds, reptiles, and amphibians. Therefore, we excluded case studies of plants and invertebrates from the Global Re-Introduction Perspective Series. In addition, we removed 3 case studies that did not include a translocation of a single individual.
Reproducibility	To verify the reproducibility of our research, we will make our dataset public.
Randomization	Randomization was not relevant to our study. For sample size considerations, we decided to use all case studies possible.

Blinding	Blinding was not relevant to our study. All case studies were already recorded and we only extracted the relevant information for our study.

Did the study involve field work? ☐ Yes ☒ No

Reporting for specific materials, systems and methods

We require information from authors about some types of materials, experimental systems and methods used in many studies. Here, indicate whether each material, system or method listed is relevant to your study. If you are not sure if a list item applies to your research, read the appropriate section before selecting a response.

Materials & experimental systems

n/a	Involved in the study
☒	☐ Antibodies
☒	☐ Eukaryotic cell lines
☒	☐ Palaeontology and archaeology
☒	☐ Animals and other organisms
☒	☐ Clinical data
☒	☐ Dual use research of concern

Methods

n/a	Involved in the study
☒	☐ ChIP-seq
☒	☐ Flow cytometry
☒	☐ MRI-based neuroimaging
